# The Plasmodesmata-Located β-1,3-Glucanase Enzyme PdBG4 Regulates Trichomes Growth in *Arabidopsis thaliana*

**DOI:** 10.3390/cells11182856

**Published:** 2022-09-13

**Authors:** Yijie Fan, Shuangshuang Lin, Tongtong Li, Fengjuan Shi, Guangyao Shan, Fanchang Zeng

**Affiliations:** State Key Laboratory of Crop Biology, Shandong Agricultural University, Tai’an 271018, China

**Keywords:** trichomes, plasmodesmata, β-1,3-glucanase, intercellular permeability

## Abstract

Intercellular material transport and information transmission in plants are carried out through the plasmodesmata (PD). The amount of callose around the PD controls channel permeability. In plants, β-1,3-glucanase can degrade callose and affect plant growth and development. In this study, the gene producing PD-localized β-1,3-glucanase and regulating the leaf trichomes is identified and named *PdBG4*. Based on functional analysis through a series of genetic manipulation assays, we found that the high expression of *PdBG4* was associated with strong PD permeability and short *Arabidopsis thaliana* leaf trichomes. Conversely, the low expression of *PdBG4* correlated with weak PD permeability and long *Arabidopsis thaliana* leaf trichomes. This study revealed that the *PdBG4* gene negatively modulates leaf trichome growth and development by regulating PD permeability.

## 1. Introduction

Trichomes are epidermal products on the surface of aerial organs of terrestrial plants. Branched or unbranched, unicellular or multicellular, and glandular or non-glandular are several types of trichomes. They can help plants adapt to many abiotic and biotic stresses, such as reducing transpiration, regulating surface temperature, improving plant frost resistance, isolating herbivores (such as insects), and shielding from ultraviolet radiation [1,2,3]. Glandular secretory trichomes (GSTs) usually synthesize and secrete many commercially valuable plant secondary metabolites and are, therefore, regarded as a “chemical factory” [4], such as artemisinin, the sesquiterpene lactone accumulated in the glandular hairs of *Artemisia annua*, which can be used to treat malaria [5,6]. The unicellular, non-glandular trichomes of *A. thaliana* are an excellent experimental system for studying the molecular mechanisms of cell differentiation and pattern formation in plants. *Arabidopsis thaliana* leaf trichomes are a kind of branched, unicellular, non-glandular epidermal hair.

Plasmodesmata (PD) are microscopic channels which traverse the cell walls of plant cells, enabling transport and communication between them. Nutrients, proteins, RNA, viruses, and metabolites can be transported between cells through the PD, which plays a vital role in regulating plant development and responding to environmental stimuli [7,8,9,10,11,12]. The turnover of callose around PD leads to the opening and closing of PD channels, which affects the transport of intercellular substances, including not only targeted molecules but also non-targeted molecules [13,14,15,16,17,18]. Callose is involved in many different stages of plant development [14,19,20,21], and it also responds to biotic and abiotic stresses [13,18,22,23,24,25].

The synthesis of callose is regulated by callose synthase (CALS) or the glucan synthase-like enzyme (GSL), and its degradation is regulated by β-1,3-glucanase (BG), as the two enzymes are antagonistic [26]. *Arabidopsis thaliana* β-1,3-glucanase 1 (PdBG1), β-1,3-glucanase 2 (PdBG2), and β-1,3-glucanase 3 (PdBG3) are located in PD; PdBG1, and PdBG2 play a role in callose degradation [27].

In order to discover more PdBG family members in Arabidopsis, in this study, we identify a β-1,3-glucanase candidate gene with homologies to PdBGs named *PdBG4*. The subcellular localization and related function are investigated here. This study is to analyze a new gene related to the regulation of PD that affects growth and development in plant.

## 2. Materials and Methods

### 2.1. Plant Material

*Nicotiana benthamiana* and *N. tabacum* were used for subcellular localization and subcellular co-localization, respectively. The *Arabidopsis thaliana* ecotype *Columbia* was used for gene transformation. *A. thaliana* was grown at 22 °C with 65% humidity and 16 h light: 8 h dark with white fluorescent light conditions in a growth room. *Nicotiana benthamiana* and *N. tabacum* plants were grown at 24 °C in long-day conditions (16 h light:8 h dark and 65% humidity with white fluorescent light) in a growth room.

### 2.2. Sequence Alignment and Phylogenetic Analysis

Multiple alignments and phylogenetic analyses based on protein sequences of BG were performed using ClustalW and Molecular and Evolution Genetic Analysis software version 6 (MEGA6) [28] and were inferred using the neighbor-joining method. Sequence alignment was performed based on BG protein sequences using DNAMAN software version 6.0 (Lynnon Biosoft, San Ramon, CA, USA). Both analyses were based on their default values.

### 2.3. Vector Construction and Plant Transformation

The specific fragment and full-length cDNA of *AtPdBG4* were amplified (primer sequences shown in Supplemental Table S1) and inserted into plasmid vectors pB7GWIWG2(II) and pEarlyGate 203, respectively, to construct RNAi and OE structures. The RNAi and OE structures of *P35S:AtPdBG4* were generated by an LR reaction. The target plasmids were introduced into *A. thaliana* via *Agrobacterium tumefaciens* (LBA4404 strain).

### 2.4. Subcellular Localization and Subcellular Co-Localization

Based on the Gateway LR reaction with the Clonase™ II enzyme mix (Thermo Fisher, Invitrogen™, Carlsbad, CA, USA), pGWB454 and pGWB4 were used to construct subcellular localization GFP and subcellular co-localization RFP fusion proteins, respectively. Subcellular localization is the construction of the full-length cDNA of *AtPdBG4* without a stop codon onto pGWB4, transforming it into 5-week-old *N. benthamiana* leaves via agroinfiltration [29] and observing the green fluorescence with a confocal laser microscope (Zeiss, Germany, lsm880). Subcellular co-localization is the construction of the full-length cDNA of *AtPdBG4* without a stop codon into pGWB454, transforming it into CMV MP-GFP transgenic *N. tabacum* via agroinfiltration, observing the red fluorescence through a confocal laser microscope, and analyzing the signal colocalization with the green fluorescence of CMV MP-GFP. Fiji (Fiji is just ImageJ, https://imagej.net/Fiji/Downloads, 27 August 2022) [30] was used to perform a correlation analysis and to plot scatterplots of pixel signal intensities for subcellular co-localization.

### 2.5. RT-PCR and qRT-PCR

Arabidopsis rosette leaves were removed from newly flowering Arabidopsis, immediately frozen in liquid nitrogen, and stored at −80 °C before use. According to the manufacturer’s instructions, the total RNA was extracted from *Arabidopsis thaliana* using an RNAprep Pure Polysaccharide Polyphenols Total Plant RNA Extraction Kit (Tiangen, Beijing, China, DP441). EasyScript^®^ One-Step gDNA Removal and cDNA Synthesis SuperMix (Transgen, Beijing, China, AE311) and oligo(DT) primers were used for complementary DNA (cDNA) synthesis. The primers used in RT-PCR and qRT-PCR are shown in Supplemental Table S1. qRT-PCR was performed using UltraSYBR Mixture (Low ROX) (CW2601, CWBIO).

### 2.6. Particle Bombardment and GFP Mobility Assays

The GFP was introduced into epidermal cells of 3-week-old *A. thaliana* leaves by microprojectile bombardment, as described by previous studies [23,31,32]. Five to eight leaves were taken from wild-type, OE, RNAi, and mutant plants and placed on agar plates for each bombardment. The lower epidermal cells of leaves were analyzed with a confocal laser scanning microscope (CLSM). The GFP fluorescence was excited with a 488 nm argon laser. Statistical analysis was performed using one-way ANOVA. Boxplots were performed using the ggplot2 package.

### 2.7. Callose Staining and Quantification

Arabidopsis leaves of wild-type, mutant, Atpdbg4 OE, and atpdbg4 RNAi lines, as described by a previous study [23], were cut into 6 × 4 mm leaf sections, cultured on 1% agar dishes for 2 days, and then soaked in 85% ethanol overnight until bleaching. The leaves were transferred into a mixture of 0.1% aniline blue double-distilled water and 1 m glycine with pH 9.5 and a volume ratio of 2:3 for 5 h. The fluorescence signal of aniline blue was observed and photographed under ultraviolet conditions. Fiji (Fiji is just ImageJ, https://imagej.net/Fiji/Downloads, 27 August 2022) [30] was used to perform pixel signal intensity quantification. One-way ANOVA was used for significance analysis.

### 2.8. Accession Numbers

Sequence data from this article can be found at The Arabidopsis Information Resource (TAIR) (https://arabidopsis.org/, 27 August 2022) under accession number *AtPdBG4* (At1G11820).

## 3. Results and Discussion

### 3.1. PdBG4 Located on PD in Arabidopsis thaliana

**Figure 1:**
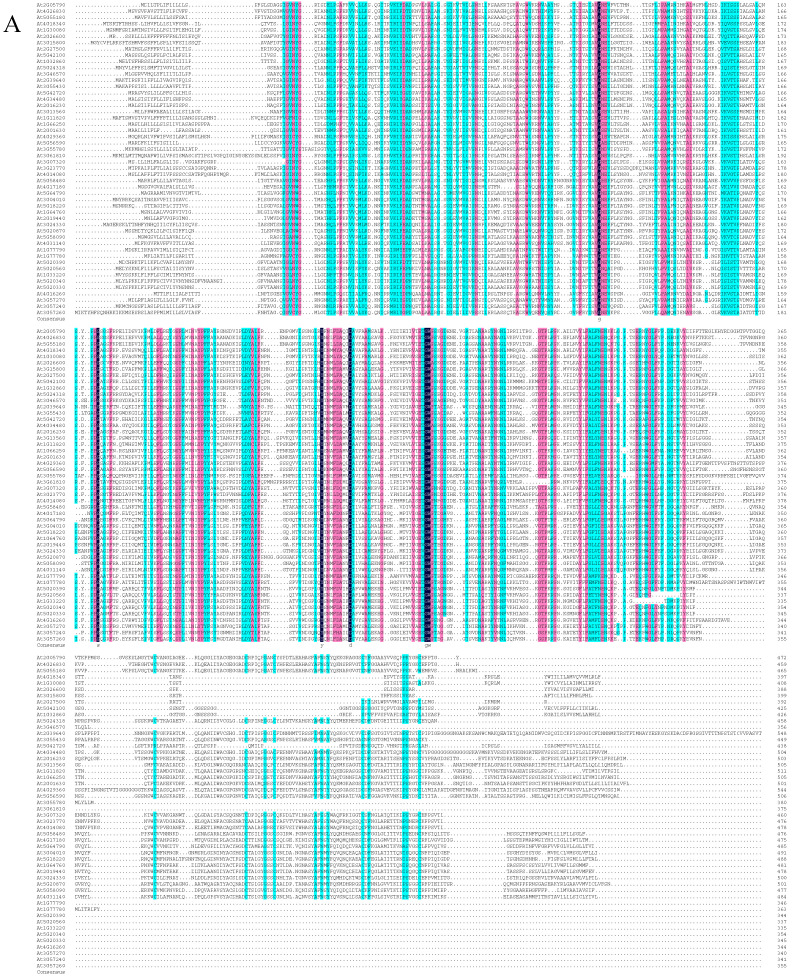

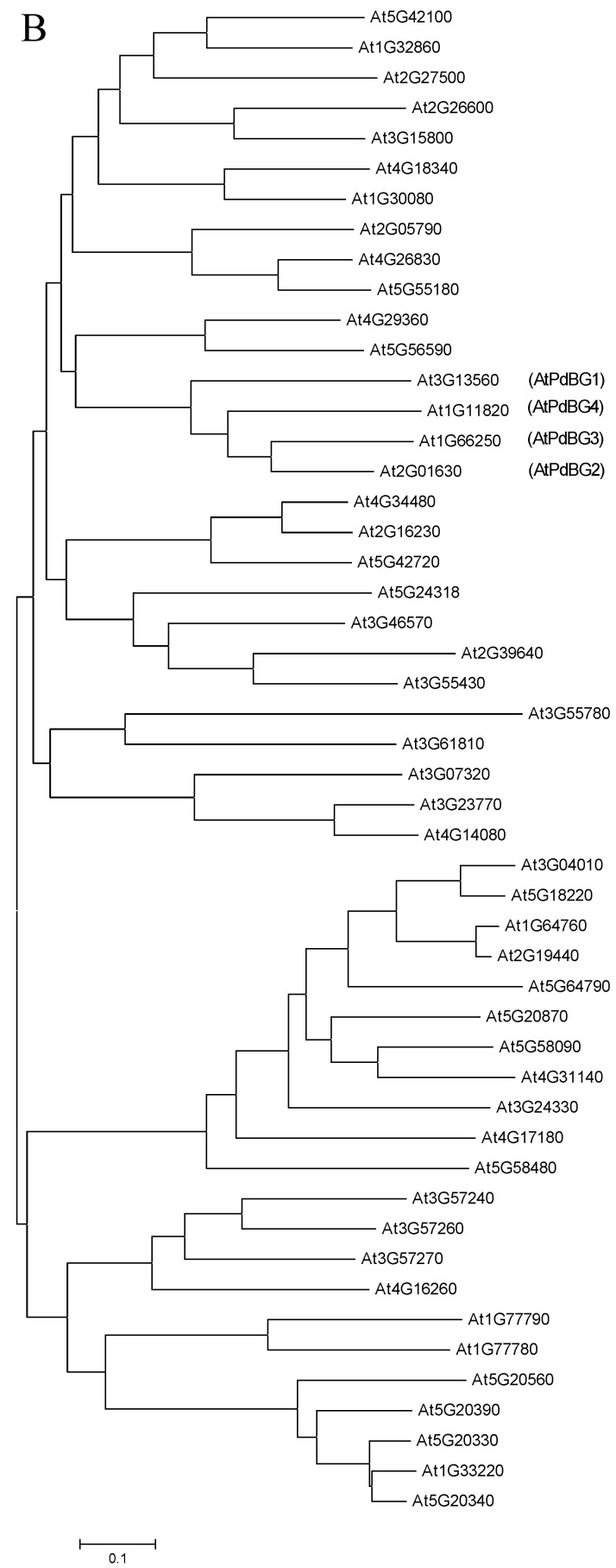
Sequence alignment and phylogenetic analysis. (**A**) Sequence similarity of β-1,3-glucanases among *Arabidopsis thaliana* BG family members Dark blue indicates that the homology level is 100%, pink indicates that the homology level ≥75%, and blue indicates that the homology level ≥50%. (**B**) Phylogenetic analysis of β-1,3-glucanases based on the amino acid sequences of *Arabidopsis thaliana* BG family members. The scale bar represents genetic distance.

**Figure 2:**
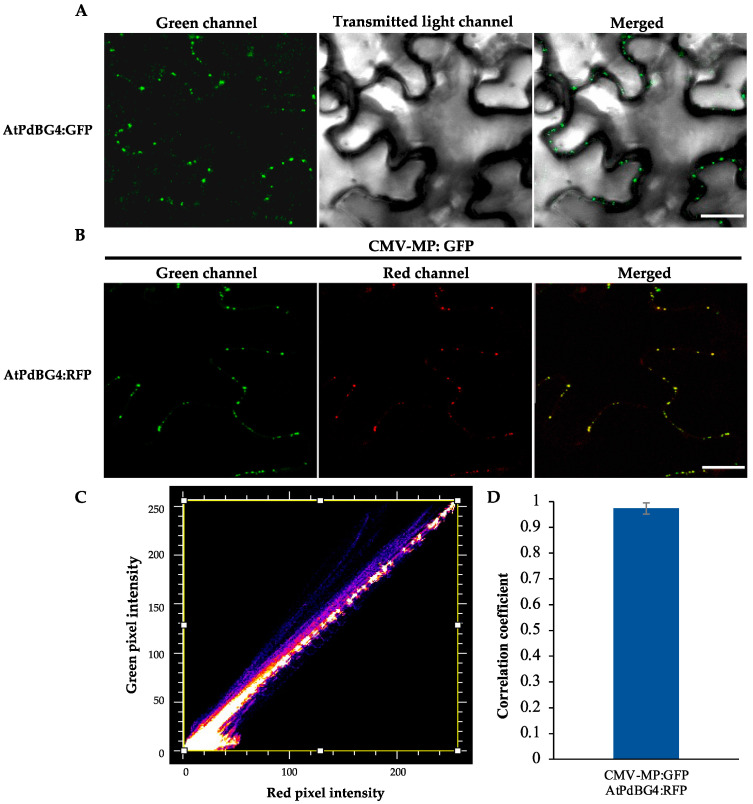
PdBG4 subcellular localization on PD. (**A**) Subcellular localization of AtPdBG4 fluorescence signal near the PD. (**B**) AtPdBG4 co-localized with the PD marker CMV MP-GFP. The C-terminal RFP-tagged AtPdBG4 was agroinfiltrated into the leaves of CMV MP-GFP transgenic N. tabacum plants. The yellow signal in the merged images represents the co-localization of AtPdBG4 with CMV MP-GFP. Images were obtained by CLSM. Bars = 20 µm. (**C**) Scatterplot of red and green pixel intensities of CMV MP-GFP and AtPdBG4:RFP. (**D**) Pearson correlation coefficient (PCC) of the images of green CMV MP-GFP and red AtPdBG4:RFP.

Based on the phylogenetic tree analysis (Figure 1) of the β-1,3-glucanase gene family in *Arabidopsis thaliana* [33], we found a specific clade that contained four genes, three of which encoding proteins that were previously reported to be associated with the plasmodesmata (PD). As a result, we speculated that the other one (At1G11820) was also PD-related. Therefore, we performed subcellular localization and subcellular co-localization assays on it. The subcellular localization assay (Figure 2A) showed that it was located in the PD region. The subcellular co-localization assay (Figure 2B) found that the fluorescent signal region was collocated with the PD marker CMV MP-GFP. The scatterplots of the pixel signal intensities are shown in Figure 2C, and the Pearson correlation coefficient (PCC) of the images of green CMV MP-GFP and red AtPdBG4:RFP was 0.99 (Figure 2D), indicating that the protein was located in the PD. Therefore, we named the gene PdBG4 (At1G11820, Figure 1).

### 3.2. PdBG4 Regulates the Permeability of PD and the Length of Leaf Trichome

**Figure 3:**
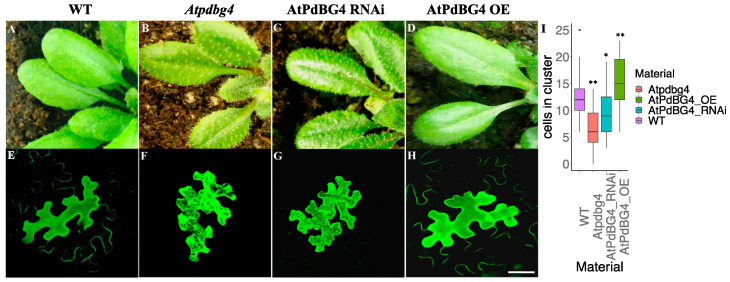
The phenotype of *AtPdBG4* transgenic *Arabidopsis thaliana* and diffusion of free GFP in leaf epidermal cells. (**A**–**D**) Silencing and overexpression of *AtPdBG4* in *A. thaliana* changed the phenotype of leaf trichomes. (**E**–**H**) Extreme representative picture of transient-expressed free GFP distribution among leaf epidermal cells in WT (**E**), *AtPdBG4* mutant (**F**), RNAi transgenic (**G**), and OE transgenic (**H**) *Arabidopsis thaliana* epidermal cells. Bar = 30 µm. (**I**) Boxplot diagram of GFP cluster sizes in WT, *Atpdbg4*, *AtPdBG4* RNAi, and *AtPdBG4* OE materials (determined by the number of cells in the cluster) 48 h after bombardment. The boxplot shows that the line in the middle of the box is the median value, and the median values of the WT, *Atpdbg4*, *AtPdBG4* RNAi, and *AtPdBG4* OE materials are 12, 6, 9, and 15, respectively. The statistical analysis was performed using one-way ANOVA based on R. * indicates *p* < 0.05; ** indicates *p* < 0.01.

We constructed *AtPdBG4* overexpression (OE) and RNA interference (RNAi) plants and ordered an *Atpdbg4* mutant (SALK_066140) to identify the effect of *AtPdBG4* gene expression changes on the phenotype of Arabidopsis. SALK_066140 was used as a mutant in this experiment. An analysis of PCR on the T-DNA line showed that the insertion in SALK_066140 disrupted the gene at 1062 bp downstream of the ATG start codon (Supplemental Figure S1A). The *AtPdBG4* RNA was not transcribed in homozygous insertion, as determined by RT-PCR (Supplemental Figure S1B). Through gene expression detection, it was found that the expression of *AtPdBG4* increased in the OE line, decreased in the RNAi line, and was not expressed in the *Atpdbg4* mutant. In contrast, the expressions of other homologous genes (*AtPdBG1*, *AtPdBG2*, and *AtPdBG3*) were not significantly different from that of the wild-type line (Supplemental Figure S1C). The *Arabidopsis thaliana* leaf trichome phenotypes in the *AtPdBG4* OE, RNAi, and mutant lines showed that high *AtPdBG4* expression was associated with short trichomes, while low *AtPdBG4* expression correlated with long trichomes (Figure 3A–D). It was obvious that the trichomes of the mutant were longer than that of the RNAi plant.

To explore the PD status by detecting intercellular permeability in transgenic *A. thaliana* plants, as reported by previous studies [23,31,32], the investigation found that *AtPdBG4* gene silencing and overexpression changed the extent of free GFP diffusion in leaf epidermal cells. After 48 h of bombardment, epidermal cells were scanned using confocal laser scanning microscopy to analyze the numbers of cells in clusters generated by free GFP movement. The numbers of counted cells were those in the presence of GFP movement around the bombarded cells (Figure 3E–H). Free GFP diffused through PD in the epidermal cells of WT *A. thaliana* leaves (Figure 3E). Free GFP diffused poorly among the leaf epidermal cells of the RNAi and mutant plants (Figure 3F,G), as well as at a lower degree in the mutant plants than in the RNAi material, and appeared to diffuse strongly among the epidermal cells of *AtPdDBG4* OE plants (Figure 3H). In *AtPdBG4* RNAi, OE, and mutant plants, the size exclusion limit (SEL) of PD was different, which changed the permeability of PD and the extent of free GFP diffusion, thus changing the number of cells with green fluorescent protein around the bombarded cells. One-way ANOVA analyses of free GFP diffusion in leaf epidermal cells revealed a significant difference between WT and other mutant or transgenic plants (*p* < 0.05). Aniline blue was used for staining the callose of Arabidopsis leaves, and the mutant line had the highest callose accumulation, followed by the RNAi line, while the *AtPdBG4* OE line had the lowest callose content (Supplemental Figure S2). One-way ANOVA revealed that there were significant differences between WT and other mutant or transgenic plants. These results suggest that the *AtPdBG4* gene modulated PD permeability through the degradation of callose, regulating the length of epidermal hair. High gene expression promoted the degradation of callose, leading to strong PD permeability and short leaf trichomes. In contrast, low gene expression reduced the degradation of callose, resulting in weak PD permeability and long leaf trichomes.

### 3.3. Discussion

The β-1,3-glucanase gene family is a complex, large gene family involved in plant pathogen defense, pollen development and pollen tube growth, cell division, stress response, the regulation of plasmodesmal signal transmission, seed germination and maturation, etc. [34,35,36,37,38,39]. For example, some of the β-1,3-glucanase family genes are involved in the degradation of the callose wall surrounding the tetrad before microspores are released into the anther locule. It also involves the dissolution of callose in the stylar matrix during the growth of pollen tubes, which is applicable to both glucanase expressed in the style [40] and glucanase found in pollen grains [41,42]. A number of β-1,3-glucanase family genes are abundantly expressed in many tissues and organs, such as flowers, seeds, and stem tips, and are widely expressed in the whole plant. These genes may participate in constitutive biological processes, such as cell division and cell wall remodeling [43,44,45].

Previous studies have noted that the degradation and removal of callose play a role in active meristem and cell division [43,44,45]. Series β-1,3-glucanase family genes are highly expressed in roots and leaves, and these genes show a significant response to fungal pathogens. These genes may play a common role in response to stress and pathogen attack [33]. Based on the available public Arabidopsis RNA-seq database (ARS) (http://ipf.sustech.edu.cn/pub/athrna/, 27 August 2022) [46], the *AtPdBG4* gene is associated with leaf development. According to previous research, it is speculated that the *PdBG4* gene may also play a role in stress responses or responses to pathogens. Therefore, we can explore whether the PdBG4 gene participates in these two processes in the future.

Our study provided evidence that the previously uncharacterized Arabidopsis β-1,3-glucanase protein was localized in the plasmodesmata and affected the growth of trichomes. Changing the level of this enzyme could provide a strategy to change the length of trichomes, which may improve the protection they provide or produce valuable trichome metabolites. As shown in previous studies, PdBG1, PdBG2, and PdBG3, as the most homologous proteins of PdBG4 in the same evolutionary clade, are located on the PD. It was reported that PdBG1, PdBG2, and PdBG3 were necessary and sufficient to regulate intercellular metastasis during the formation of lateral root primordia (LRP) [27]. However, in the mutant and transgenic materials of the *PdBG4* gene that we identified, there were no differences found between the wild-type and *PdBG4* transgenic or mutant plants in the formation of LRP. Based on the public Arabidopsis RNA-seq database ARS, *PdBG4* was found to be more highly expressed in leaves than in roots compared with *PdBG1*, *PdBG2*, and *PdBG3*. These results indicated the functional differentiation during evolution between the *PdBG4* gene and other PdBG homologous genes. The reasons for the functional differentiation of PdBG are still unclear, and physiological and molecular regulatory mechanisms underlying the complex developmental process mediated by PdBG still need to be further studied.

## 4. Conclusions

In this study, we identified intercellular plasmodesmata located in the β-1,3-glucanase enzyme PdBG4. This gene regulated the growth and development of leaf epidermal hairs by regulating the permeability of the plasmodesmata in Arabidopsis. The high expression of *PdBG4* promoted the degradation of callose, leading to strong PD permeability and short leaf epidermal hair. On the contrary, the low expression of *PdBG4* reduced the degradation of callose, resulting in weak PD permeability and long leaf trichomes. This regular pattern could be practically used to control the formation of epidermal hair to improve the tolerance of plants to biological and abiotic stimuli. This study provides new insight into plant trichome growth and development regulation and important candidate gene resources in trichome function, which can be applied in plant resistance breeding using biotechnologies such as CRISPR/Cas9.

## Data Availability

All relevant data can be found and are available within the manuscript and supporting information.

## Supplementary Materials

The following supporting information can be downloaded at: https://www.mdpi.com/article/10.3390/cells11182856/s1. Table S1. Primers in this manuscript. Figure S1. Identification of *AtPdBG4* T-DNA insertion line and quantitative detection of gene expression in transgenic and mutant materials. (**A**) Schematic model of T-DNA insertion in the mutant. The number indicates the location of the inserted coding sequence. (**B**) The upper panel shows the amplification of the coding sequence of *AtPdBG4* and no amplification in the homozygous mutant by RT-PCR. The lower panel shows the amplification of the control gene *actin2*. (**C**) Quantitative real-time PCR detection of AtPdBG for wild-type, *Atpdbg4* (SALK_066140), *AtPdBG4* RNAi, and *AtPdBG4* OE materials. *AtPdBG4* gene mutation, RNA interference, and overexpression only affected the expression of the *AtPdBG4* gene but did not affect the expressions of *AtPdBG1*, *AtPdBG2*, and *AtPdBG3*. Student’s *t*-test was used to perform statistical analysis; ** indicates *p* < 0.01. Figure S2. Aniline-blue-stained callose present around plasmodesmata. (**A**–**D**) Aniline blue staining of callose in leaves of Arabidopsis WT, *Atpdbg4*, AtPdBG4 RNAi, and AtPdBG4 OE lines, respectively. Bar = 10 µm. (**E**) Aniline blue fluorescent pixel intensity. ImageJ was used to perform pixel signal intensity quantification, and the statistical analysis was performed using one-way ANOVA. Five leaf discs from three plants were analyzed for each experiment. * indicates *p* < 0.05; ** indicates *p* < 0.01.
