# Supplementary material for: The Plasmodesmata-Located β-1,3-Glucanase Enzyme PdBG4 Regulates Trichomes Growth in Arabidopsis thaliana
Source: Cells. 2022 Sep 13;11(18):2856. doi: 10.3390/cells11182856 (PMC9496821; doi:10.3390/cells11182856)
Supplement: Supplementary file 1 [file cells-11-02856-s001.zip › cells-1922256-supplementary.pdf]

Table S1. Primers in this manuscript.

Primer	5′-3′
AtPdBG4-OE-F	ATGGCTTTCACTAGCATGGTTTC
AtPdBG4-OE-R	TTACAATATGGAAAATGTTACAAGAAT
AtPdBG4-RNAi-F	GGATTGGGCTTGTGGACCTG
AtPdBG4-RNAi-R	CCTATTTCCGACCTTCTTGC
AtPdBG4-GFP/RFP-F	ATGGCTTTCACTAGCATGGTTTC
AtPdBG4-GFP/RFP-R	CAATATGGAAAATGTTACAAGAATC
AtPdBG4-RT-F	GTTATTCTATGGGAACTCTACAC
AtPdBG4-RT-R	TTCACATTATTCGGCTGGT
AtPdBG1-qRT-F	GTGGCTCTAGCTCCGCAG
AtPdBG1-qRT-R	CGCAGTTAGCTCGGCCTT
AtPdBG2-qRT-F	CCTTCTGAGCACGACGCA
AtPdBG2-qRT-R	CTGCAGTTCCCGGGTGTT
AtPdBG3-qRT-F	CGCTGATCCCGGGTTGTT
AtPdBG3-qRT-R	CCCAATTCGCTGCGGTTG
AtPdBG4-qRT-F	AGCTGCATTTTCCCGGGA
AtPdBG4-qRT-R	AAGTAGCTTCACCCGCGG
actin-2F	TCAGATGCCCAGAAGTCTTGTTCC
actin-2R	CCGTACAGATCCTTCCTGATATCC

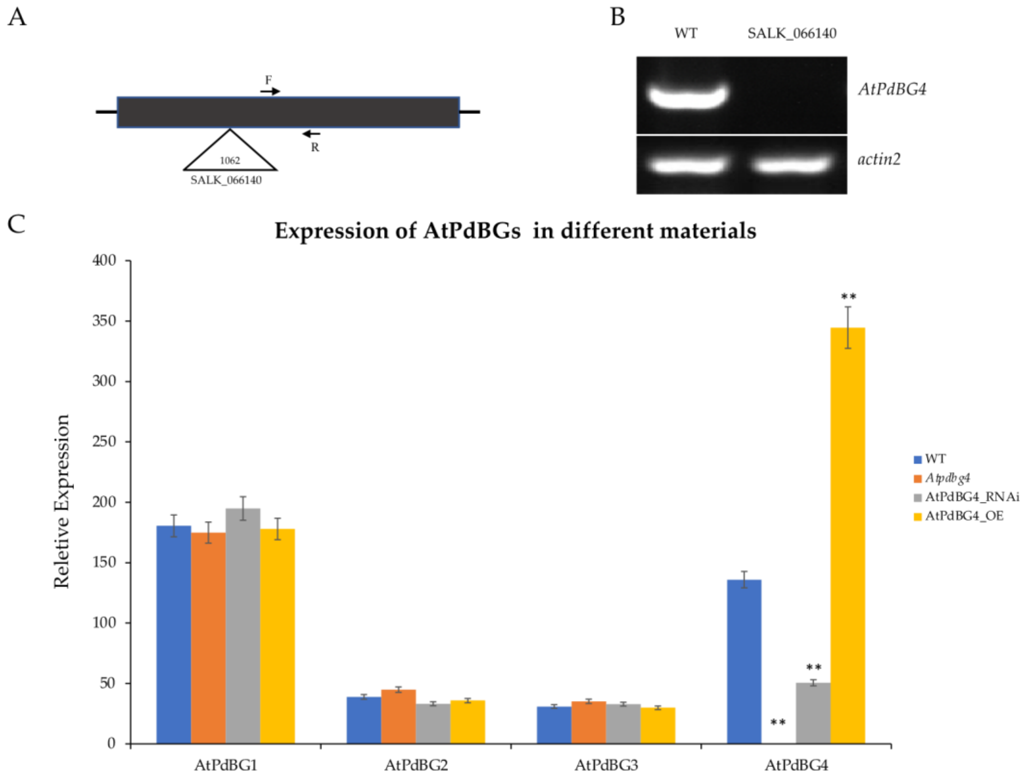

Figure S1. Identification of *AtPdBG4* T-DNA insertion line and quantitative detection of gene expression in transgenic and mutant materials. (**A**) Schematic model of T-DNA insertion in the mutant. The number indicates the location of the inserted coding sequence. (**B**) The upper panel shows the amplification of the coding sequence of *AtPdBG4* and no amplification in the homozygous

mutant by RT-PCR. The lower panel shows the amplification of the control gene *actin2*. (**C**) Quantitative real-time PCR detection of AtPdBGs for wildtype, *Atpdbg4* (SALK_066140), *AtPdBG4* RNAi, and *AtPdBG4* OE materials. *AtPdBG4* gene mutation, RNA interference, and overexpression only affected the expression of the *AtPdBG4* gene but did not affect the expressions of *AtPdBG1*, *AtPdBG2*, and *AtPdBG3*. Student's t-test was used for statistical analysis;, ** indicates $p < 0.01$.

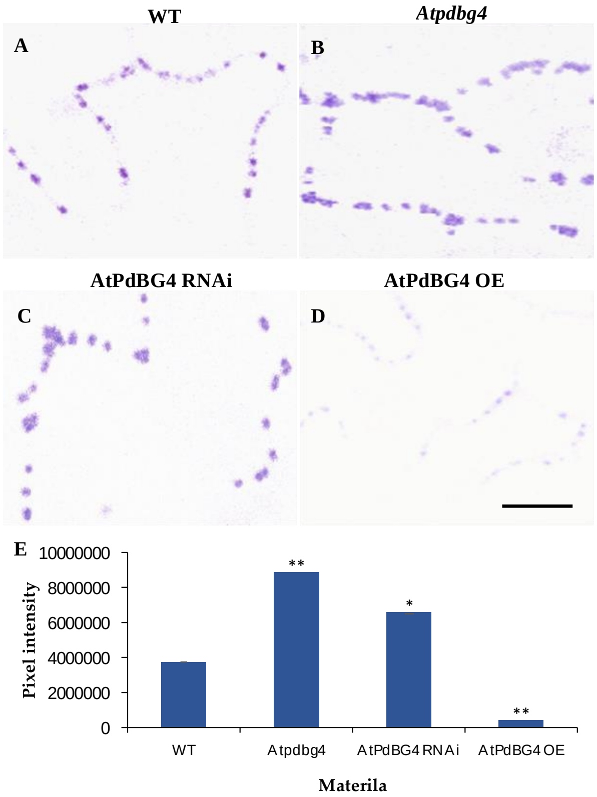

Figure S2. Aniline-blue-stained callose present around plasmodesmata. (**A-D**) Aniline blue staining of callose in leaves of Arabidopsis WT, *Atpdbg4*, AtPdBG4 RNAi, and AtPdBG4 OE lines, respectively. Bar = 10 μm. (**E**) Aniline blue fluorescent pixel intensity. ImageJ was used to perform pixel signal intensity quantification, and the statistical analysis was performed using one-way ANOVA. Five leaf discs from three plants were analyzed for each experiment. * indicates $p < 0.05$;, ** indicates $p < 0.01$.
